# Association of salivary steroid hormones and their ratios with time-domain heart rate variability indices in healthy individuals

**DOI:** 10.5937/jomb0-26045

**Published:** 2021-03-12

**Authors:** Eglė Mazgelytė, Gintaras Chomentauskas, Edita Dereškevičiūtė, Virginija Rekienė, Audronė Jakaitienė, Tomas Petrėnas, Jurgita Songailienė, Algirdas Utkus, Kučinskienė Zita Aušrelė, Dovilė Karčiauskaitė

**Affiliations:** 1 Vilnius University, Faculty of Medicine, Institute of Biomedical Sciences, Department of Physiology, Biochemistry, Microbiology, and Laboratory Medicine, Vilnius, Lithuania; 2 Human Study Center, Vilnius, Lithuania; 3 Vilnius University, Faculty of Medicine, Institute of Biomedical Sciences, Department of Human and Medical Genetics, Vilnius, Lithuania

**Keywords:** autonomic nervous system, heart rate variability, hypothalamic-pituitary-adrenal axis, steroid hormones, stress biomarkers, autonomni nervni sistem, varijabilnost srčanog ritma, hipotalamusno-hipofizno-adrenalna osa, steroidni hormoni, biomarkeri stresa

## Abstract

**Background:**

Stress system consists of the hypothalamicpituitary-adrenal (HPA) axis and the locus caeruleus/norepinephrine-autonomic nervous system (ANS). Traditionally, HPA axis activity is evaluated by measuring its end-product cortisol, while the activity of ANS is assessed using heart rate variability (HRV) indices. Alterations in cortisol levels and HRV measures during laboratory-based stress tasks were extensively studied in previous research. However, scarce data exist on the associations of HRV measures with the levels of other adrenal steroid hormones under baseline conditions. Thus, we aimed to evaluate the activity of the HPA axis by measuring salivary cortisol, cortisone, dehydroepiandrosterone (DHEA) levels, and their ratios and to examine its association with HRV measures in a sample of healthy young and middle-aged adults.

**Methods:**

For each participant (n=40), three data collection sessions taking place at the same time of the day were scheduled within five working days. Participants completed a self-reported questionnaire on sociodemographic and lifestyle characteristics, filled out t h e Perceived Stress Scale and State-Trait Anxiety Inventory. Also, saliva samples were collected, and physiological measures, including resting HR and HRV, were recorded during three data collection sessions.

**Results:**

Statistically significant associations between diminished parasympathetic vagal tone evaluated by time domain HRV measures and higher salivary cortisol, lower DHEA levels, as well as decreased DHEA to cortisol ratio, were found. Also, physiological stress indicators (i.e., HRV) showed greater intraindividual stability compared with biochemical biomarkers (i.e., salivary steroid hormones) within five days.

**Conclusions:**

Our findings suggest that both cortisol and DHEA mediate the link between two stress-sensitive homeostatic systems.

## Abstract (sr)

**Uvod:**

Sistem stresa se sastoji od hipotalamusno-hipofiznoadrenalne (HPA) ose i plavog jedra/norepinefrina - autonomnog nervnog sistema (ANS). Tradicionalno, aktivnost HPA ose se procenjuje merenjem kortizola u krajnjem produktu, dok se aktivnost ANS procenjuje pomoću indeksa promenljivosti otkucaja srca (HRV). Promene nivoa kortizola i HRV merene tokom stresnih zadataka u laboratorijskim uslovima su detaljno proučavane u prethodnim istraživanjima. Međutim, postoje nedovoljni podaci o povezanosti merenja HRV-a sa nivoima ostalih nadbubrežnih steroidnih hormona u osnovnom stanju. Stoga smo želeli da procenimo aktivnost HPA ose merenjem nivoa kortizola iz pljuvačke, kortizona i dehidroepiandrosterona (DHEA) i da ispitamo njihovu povezanost sa HRV merenjima na uzorku zdravih mladih ljudi i ljudi srednjih godina.

**Metode:**

Za svakog učesnika (n=40) su zakazane tri sesije prikupljanja podataka u isto doba dana tokom pet dana. Učesnici su samostalno popunili upitnik o sociodemografskim i životnim karakteristikama, ispunili su Percipiranu skalu stresa i Upitnik o anksioznosti kao stanja i osobine ličnosti. Takođe, prikupljeni su uzorci pljuvačke i urađena su fiziološka merenja, uključujući HR i HRV u mirovanju tokom tri sesije prikupljanja podataka.

**Rezultati:**

Otkrivene su statistički značajne veze između smanjenog parasimpatičkog vagalnog tona procenjenog vremenskim domenom HRV i višeg nivoa kortizola u pljuvački, nižeg nivoa DHEA, kao i smanjenog racija DHEA i kortizola. Takođe, pokazatelji fiziološkog stresa (tj. HRV) pokazali su veću intraindividualnu stabilnost u poređenju sa biohemijskim biomarkerima (tj. steroidnim hormonima pljuvačke) u periodu od pet dana.

**Zaključak:**

Naša otkrića sugerišu da kortizol i DHEA posreduju u vezi između dva homeostatska sistema osetljiva na stres.

## Introduction

Stress can be defined as a nonspecific response of the body to any demand made on it. It is proposed that chronic stress is associated with the prevalence of depression, anxiety disorders, cardiovascular disease, type 2 diabetes, osteoporosis, and metabolic syndrome [1]. Thus, the importance of accurate and reliable stress assessment techniques is unquestionable. Two major neural systems, including hypothalamicpituitary-adrenal (HPA) axis and autonomic nervous system (ANS), are involved in the adaptation of the organism to stressful situations [1] [2] [3]. Determination of HPA axis end-product cortisol levels in different biological matrices (e.g., blood serum, saliva, urine, hair, nails) has been frequently used to evaluate HPA axis activity, while ANS is usually assessed by measuring time and frequency-domain heart rate variability indices (HRV) [2] [3] [4]. Previous research was mainly done on changes in salivary cortisol (saCORT) concentration and HRV measures in response to acute laboratory stress. Results showed that laboratory stressors evoked increased cortisol secretion, raised heart rate (HR), and diminished HRV representing higher HPA axis activity and altered sympathovagal balance under the influence of acute stress [5] [6]. However, most of the studies examining the relationship between changes in HRV indices and cortisol levels in response to laboratory-based stress tasks found nonsignificant results [7] [8]. Along this line, there is some evidence that significant alterations in HRV occurs during the anticipation of a stressful event, and these changes are associated with interindividual differences in stress-induced cortisol secretion [6]. Despite extensive research, very little is known about interconnections between the HPA axis and ANS under baseline conditions. The present study was dedicated to evaluating the activity of HPA system by measuring singlepoint salivary cortisol, cortisone, dehydroepiandrosterone (DHEA) levels, and their ratios and to examine its association with ultra-short-term time-domain heart rate variability measures such as the square root of the mean squared differences of successive R-R intervals (RMSSD) and the percentage of R-R intervals with more than 50-ms variation (pNN50). We also aimed to compare the intraindividual stability of biochemical (salivary steroid hormones and their ratios), and physiological (resting HR and HRV measures) stress indicators during three specimen collection sessions scheduled within five working days.

## Materials and Methods

### Study population

Forty apparently healthy volunteers (age 21–53 years), comprising 26 (65%) women and 14 (35%) men, participated in the study. Participants were recruited in Human Study Center via pre-registration forms. Each enrolled individual was contacted by experimenters by phone. Participants were excluded for medical conditions, including chronic heart disease, metabolic and endocrine disorders, as well as mental diseases. Table 1 reports sociodemographic, lifestyle characteristics, and psychological measures of the study sample.

**Table 1:** Sociodemographic, lifestyle characteristics, and psychological measures of the study sample. Note: n=40 for all the variables except for Smoking status (n=38), Exposure to environmental tobacco smoke (n=38), Physical activity at work (n=38), and Leisure-time physical activity (n=38).

Variable	Mean ± SD or N(%)
Gender	
Women	26 (35.0)
Men	14 (65.0)
Age (Years)	34.8±7.5
**Marital status**	
Single	16 (40.0)
Married	21 (52.5)
Divorced	3 (7.5)
**Education**	
Secondary	1 (2.5)
Tertiary	39 (97.5)
**Self-reported general health status**	
Excellent	12 (30.0)
Good	25 (62.5)
Moderate	3 (7.5)
Poor	0
Very bad	0
**Smoking status**	
Non-smoker	30 (79.0)
Moderate smoker	3 (7.9)
Heavy smoker	5 (13.1)
**Exposure to environmental tobacco smoke**	
No	31 (81.6)
Yes	7 (18.4)
**Physical activity at work**	
Inactive	33 (86.8)
Active	5 (13.2)
**Leisure time physical activity**	
Inactive	12 (31.6)
Active	26 (68.4)
**Psychological measures**	
Perceived Stress Scale (PSS) score	16.45 ± 5.96
Spielberger State-Trait Anxiety Inventory-Trait Form (STAI-T) score	40.13 ± 6.06
Spielberger State-Trait Anxiety Inventory-State Form (STAI-S) score	36.28 ± 11.48

The participants provided written informed consent before entering the study. The study protocol was approved by the Lithuanian Bioethics Committee (No. 2019/5-1135-626). For each participant, three data collection sessions taking place at the same time of the day were scheduled within five working days, although the time of day varied between individuals. All specimens were collected during June and July 2019. The subjects were asked to refrain from alcohol consumption for 48 hours, intense exercise for 24 hours, eating, drinking (except water), smoking and brushing their teeth or using dental floss for 1 hour before each data collection session. Saliva samples were collected after 5 minutes of resting in a sitting position.

Afterwards, a high-frequency infrared light sensor was placed on the subjects' left earlobe, and recording of HR and HRV measures was started. HR and HRV measurements were performed according to the guidelines from the Society for Psycho physiological Research [9]. The measurements were recorded for 1 minute in a sitting position with knees at a 90° angle, both feet flat on the floor and hands placed on thighs. The participants were asked to stay seated without speaking or making any movements during the recording period. Since previous research [10] showed that resting-state HRV is recorded best under spontaneous breathing conditions, respiration frequency was not controlled in the current study. Also, on the first data collection session, participants completed a self-reported questionnaire on sociodemographic and lifestyle characteristics, filled out the Perceived Stress Scale (PSS), and the State-Trait Anxiety Inventory (STAI).

### Perceived stress and anxiety measures

PSS was used as a measure of the degree to which situations in subjects' life are appraised as stressful. The 10-item version indicated participants' stress perception over the past month. The participants were asked to rate each item on a Likert-type response scale ranging from 1 = never to 5 = very often. A higher overall score indicates a greater perceived stress level.

State and Trait Anxiety subscales of the STAI were used as a subjective measure of anxiety. The S-Anxiety scale consisted of twenty statements that evaluate how respondents feel »right now, at this moment.« The T-Anxiety scale consisted of twenty statements that assess how subjects generally feel. Each STAI score ranges from 20 to 80, with higher scores indicating greater state and trait anxiety levels [11].

### Salivary steroids analysis

The samples were collected using Salivette^®^ (Sarstedt, Rommelsdorft, Germany) devices. Saliva samples were stored at -80°C until the analysis. The samples were treated in the sequence of centrifugation for 10 min at 4000 rpm, liquid-liquid extraction with ethyl acetate, and resuspension in methanol/water containing 0.1% formic acid in a ratio of 50:50 (v/v). The chromatographic separation was performed on the ultra-high performance liquid chromatography (UHPLC) system, which consisted of two Shimadzu LC-30AD binary pumps, a Shimadzu SIL-30AC autosampler and a Shimadzu C T O-20AC column oven (Shimadzu Corporation, Kyoto, Japan). The UHPLC was coupled with Shimadzu LCMS-8060 triple quadrupole tandem mass spectrometer equipped with a n electrospray ionization source (Shimadzu Corporation, Kyoto, Japan), which was operated in the positive ionization mode. For each analyte, two ion pairs were selected, with the most sensitive transition being used for quantification while the rest for confirmation. The UHPLC column was Poroshell 120, EC-C18, (3.0×75 mm, 2.7 μm) column (Agilent Technologies, USA). The method utilized a binary gradient with mobile phases containing methanol and water acidified with 0.1% formic acid at a flow rate of 0.5 mL/min. The injection volume was 10 μL. Data acquisition was performed by Shimadzu LabSolutions software (version 1.20).

### Heart rate variability measures

Heart rate (HR) measurements were made by high-frequency infrared light earlobe sensor, worn by each participant on the left earlobe. Each pulse duration was determined and recorded by identifying intervals between the highest blood oxygen saturation points. HR was calculated using the formula:

(1) }{}HR=\frac{60000}{HR_{durr}}

where HR_durr_ refers beat-to-beat intervals.

Heart rate variability (HRV) was evaluated using time-domain measures such as the square root of the mean of the sum of the squares of differences between adjacent NN intervals (RMSSD) and the number of interval differences of successive NN intervals greater than 50 ms divided by the total number of all NN intervals (pNN50).

### Statistical analysis

Statistical analysis was performed with R version 3.6.0. Quantitative variables are presented as mean±standard deviation (SD). For categorical variables, absolute and relative frequencies were calculated. The intraclass correlation coefficient (ICC) using a two-way mixed effect model was calculated to examine consistency among multiple measures at three different data collection days where ICC<0.40 means poor consistency, 0.40≤ICC<0.60 indicates fair consistency, 0.60≤ICC<0.75 means good consistency and 0.75≤ICC<1.00 indicates excellent consistency. The coefficient of repeated measures correlation (r_rm_) (R package rmcorr) was calculated to determine the common within-individual association for biochemical and physiological measures assessed on three occasions. r_rm_) accounts for non-independence among measures from the same individual by adjusting for variability between individuals. In r_rm_) the inter-individual variability is removed, and the best linear fit for each subject determined using regression lines with the same slope but different intercepts. Similarly, to a Pearson correlation, coefficient, r_rm_) values range from -1 to 1 and estimate the strength of the linear association between two variables [12]. The level of statistical significance was set at 0.025 for one-tailed testing.

## Results

### Psychological measures

According to the results of the PSS questionnaire, participants reported that their lives were nonstressful (25%), or they felt a moderate stress level (75%) during the last month. T-Anxiety and S-Anxiety mean scores indicated that there were no clinically significant symptoms of anxiety in our study sample, and STAI score values were comparable with STAI scores of a healthy norm population in the age of 30-59 years [11]. Further STAI score evaluation is complicated with the lack of guidelines and cut-off values for sorting participants into separate groups (with low, moderate, high anxiety level). Thus, we utilized psychological indicators to describe the overall psychological well-being of subjects without a more detailed analysis of these measures.

### Relationships between biochemical and physiological measures

**Figure 1:**
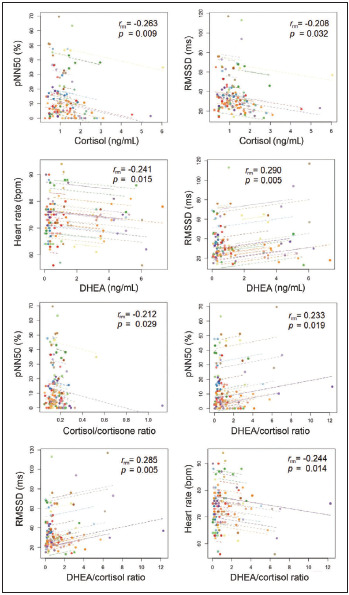
Association between biochemical and physiological stress measures across three data collection sessions. Observations from the same participant are given the same color, with corresponding lines to show the rmcorr fit for each participant.

The analysis of repeated measures correlation revealed a weak but statistically significant negative correlation between saCORT concentration and pNN50 (r_rm_=-0.263, p=0.009), and a slight tendency toward significance was observed between saCORT level and RMSSD values (r_rm_=-0.208, p=0.032). Moreover, DHEA was found to be significantly inversely related to resting HR and positively associat-ed with RMSSD (r_rm_=-0.241, p=0.015, and r_rm_=0.290, p=0.005, respectively). The association of cortisol to cortisone ratio with pNN50 values was close to being statistically significant (r_rm_=-0.212, p=0.029). Similarly, higher DHEA to cortisol ratio was significantly related to lower resting HR values and higher time-domain measures of HRV including both RMSSD and pNN50 values (r_rm_=-0.244, p=0.014, r_rm_= 0.285, p=0.005 and r_rm_=0.233, p=0.019, respectively) (Figure 1). No significant associations were found between HRV indices and cortisone level, as well as the total salivary glucocorticoid level (the sum of cortisol and cortisone concentrations).

### Consistency of biochemical and physiological measures

To determine the intraindividual stability of biochemical and physiological stress measures across three data collection sessions, we calculated the intraclass correlation coefficient (ICC). ICCs for salivary steroid hormone concentrations showed poor to fair consistency within three sessions. Among biochemical biomarkers, the highest intraindividual stability was observed for salivary cortisone concentration (ICC=0.483) and the total glucocorticoid level (ICC=0.430). In contrast to biochemical biomarkers, physiological indicators showed fear of good intraindividual stability where ICC ranged from 0.520 to 0.649 (Table 2).

**Table 2:** Intraclass correlation coefficients (ICCs) of biochemical and physiological stress indicators across three measurements.

Variable	ICC (95% CI)
**Biochemical indicators**	
Salivary cortisol concentration (ng/ml)	0.132 (-0.047, 0.346)
Salivary cortisone concentration (ng/ml)	0.483 (0.295, 0.657)
Salivary DHEA concentration (ng/ml)	0.082 (-0.090, 0.295)
Salivary cortisol/cortisone ratio	0.103 (-0.072, 0.316)
Salivary DHEA/cortisol ratio	0.000 (-0.214, 0.121)
Salivary cortisol + cortisone (ng/ml)	0.430 (0.238, 0.615)
**Physiological indicators**	
Heart rate (bpm)	0.649 (0.491,0.780)
Heart rate variability: RMSSD (ms)	0.520 (0.337,0.686)
Heart rate variability: pNN50 (%)	0.593 (0.422, 0.740)

## Discussion

The main focus of this study was to examine the association between distinct physiological and biochemical stress indicators under baseline conditions, as well as to investigate the intraindividual stability of these measures. Results showed a significant relationship between time-domain HRV indices (RMSSD and pNN50) and salivary cortisol and DHEA levels. Additionally, we have found that HR values were significantly related to DHEA concentration and DHEA to cortisol ratio. These results indicate a possible interaction between the HPA axis and ANS. The link between these two systems was also observed in the study conducted by Adlan et al. [13], who reported increases in HR and blood pressure, as well as a reduction in HRV following hydrocortisone administration in healthy young men. HRV measures, time-domain (RMSSD, pNN50), and frequency-domain (high-frequency power (HF) have been negatively associated with higher cortisol total daily output and steeper cortisol diurnal slope in the healthy pediatric population [2]. In the same study, greater cortisol awakening response was associated with higher lowfrequency to high-frequency power ratio (LF/HF), which represents reduced vagal activation. Agorastos et al. [4] reported that pharmacological HPA axis stimulation with metyrapone was associated with decreased parasympathetic activity indicated by significantly reduced RMSSD, pNN50, and HF values, while HPA axis suppression using dexamethasone had no effect on autonomic activity. Our results are in line with the previous research showing that healthy subjects with high vagal tone exhibited significantly lower evening saCORT concentrations [14]. Together, these results support the idea that under baseline conditions, there is an inhibitory role of the vagal nerve in the regulation of the HPA system. Specifically, tonic vagal inhibition of the amygdala by the prefrontal cortex (PFC) results in the attenuated HPA axis activity and decreased cortisol secretion [3] [6] [15]. On the other hand, it is proposed that higher cortisol levels act indirectly via increased serotonin reuptake and results in a breakdown of the functional connectivity between PFC and amygdala [4] [16].

Available data for the associations between salivary DHEA and cardiac autonomic regulation are very scarce but show the same trend of a positive correlation between HRV measures and DHEA or DHEA-S levels [17] [18] [19]. Although the exact mechanisms of DHEA(S) effects on sympathovagal balance are unclear, it is hypothesized that the existence of cardiac DHEA(S) receptors and the regulatory effects of DHEA(S) on sympathetic adrenal activity may serve as an essential linkage between increased HRV and higher DHEA(S) levels in blood serum or saliva samples [17] [19]. Previous research suggested that administration of DHEA in young adult males has been related to diminished activation of the amygdala and hippocampus [20]. Since stimulation of amygdala contributes to the removal of vagal inhibition on sinoatrial node, the antagonistic action of DHEA on amygdala results in decreased HR and raised HRV [3]. Another possible explanation is that DHEA may act indirectly as it serves as a precursor to sex hormones, including testosterone and estradiol [20]. Previous studies have shown that higher testosterone levels in blood serum are associated with increased HRV parameters reflecting parasympathetic dominance over sympathetic activity [19] [21]. There are theories that HRV may be controlled via cardiac receptors for testosterone. However, the existence of these receptors was confirmed in the rabbit but not in the human heart [21]. Previous studies reported positive [18], negative [19], and no [21] association between estradiol level and HRV indices reflecting vagal tone. The mechanism of how estradi-ol regulates the activity of ANS has not so far been elucidated at the molecular level. It is thought that estradiol modulates the functioning of autonomic nervous centers. Experiments performed in animal models demonstrated that increased estradiol concentration in the insular cortex was associated with a higher sympathetic activity, while direct estradiol injection into autonomic regulatory areas led to increased parasympathetic activity. Such contradictions demand further analysis to be carried out [18] [19].

Importantly, our results showed significant associations of salivary DHEA to cortisol ratio with both RMSSD and pNN50 values. This finding strongly supports the idea of the interconnection between parasympathetic vagal tone and HPA axis activity as it is proposed that DHEA to cortisol ratio is a more sensitive index of HPA axis function compared with cortisol and DHEA examined as separate biomarkers of adrenocortical activity [3] [20]. DHEA conveys protective effects by opposing the neurotoxic action of cortisol, and thus DHEA to cortisol ratio represents a net glucocorticoid activity and indicates the preferential synthesis of one hormone compared with the other [20]. In the current study, a clear tendency toward significance was observed in the association between salivary cortisol to cortisone ratio and pNN50 values. Since salivary cortisol/cortisone ratio serves as a marker of 11β-hydroxysteroid dehydrogenase type 2 (11β-HSD2) mediated conversion of the unbound fraction of cortisol to cortisone in mineralocorticoid target parotid and submandibular glands [22] [23] [24], our results indicate that downregulation of 11β-HSD2 activity may be linked to lower HRV. Since cortisol is capable of acting as mineralocorticoid receptors (MR) agonist, the main function of 11β-HSD2 in mineralocorticoid target cells is to protect non-selective MR against the binding of cortisol [22] [25] [26]. Diminished 11β-HSD2 activity results in excessive activation of MR by cortisol and hence causes lowrenin, low-aldosterone hypertension that is also referred to as »apparent mineralocorticoid excess« [26]. It was shown that blockage of MR with aldosterone antagonist, spironolactone, leads to improved time-domain HRV measures. Thus, overstimulation of MR by cortisol due to low 11β-HSD2 activity might cause reduced HRV.

Evaluation of HPA axis activity is usually restricted to its end-product cortisol concentration measurements. However, elevated HPA axis activity can also be characterized by increased aldosterone level as the adrenocorticotropic hormone is the common stimulus for cortisol and aldosterone secretion from the adrenal cortex. The importance of MR activation by aldosterone in the central nervous system is confirmed by the fact that there are certain brain areas, such as the nucleus of the solitary tract, the amygdala and the paraventricular nucleus of the hypothalamus, where MR and 11β-HSD2 are co-expressed and hence aldosterone can act specifically at MR in these regions [25]. Thus, aldosterone measurements in blood serum or saliva samples should grant with a better understanding of the relationship between the HPA axis and ANS.

To sum up, we found a significant association between HPA axis activity and sympathovagal balance in healthy non-stressed, and moderately stressed individuals. These results indicate that functional connectivity between the HPA axis and ANS under baseline conditions might be related to higher resilience to stressful situations and lower perceived stress levels. Otherwise, it is possible that disruption in the linkage between these two systems results in higher vulnerability to psychosocial stress. However, the aforementioned hypothesis should be proved in future studies recruiting physically healthy but highly stressed individuals.

Stability in steroid hormone measurements ranged from ICC=0.000 to 0.483, which implies a high degree of variability within individuals across five days. Zhang et al. also found that single-point levels of cortisol, cortisone, and their ratio showed significant day-to-day variation across three saliva collection days with an interval of one week (ICC ranged between 0.20 and 0.53). In the more recent study conducted by Ryan et al., the stability of cortisol levels at waking and bedtime was evaluated within eight days in a healthy pediatric population aged from 8 to 13. In line with our results, the latter study demonstrated high day-to-day variability in both waking and bedtime cortisol levels. Bakusic et al. reported lower day-to-day variability in morning salivary cortisone compared with cortisol levels [24]. To the best of our knowledge, no other studies assessed intraindividual stability of salivary DHEA levels and DHEA to cortisol ratio. Taken together, our results indicate that single-point salivary steroid hormone measurement can be used only as a state-like index and provide information about short-term or acute changes in HPA axis regulation.

In contrast, greater intraindividual stability was observed in physiological stress measures within three data collection sessions (ICC ranged between 0.520 and 0.649). These findings are comparable with the results of previous research. For example, Kobayashi et al. reported fair consistency (ICC= 0.58) of RMSSD values across two consecutive days of measurement in the population of 417 Japanese male students aged 20 to 29 years old. Along this line, Bertsch et al. showed good consistency (ICC ranged between 0.71 and 0.73) of both RMSSD and pNN50 values at three measurement occasions once a week under spontaneous breathing conditions in the population of 60 healthy students. To sum up, the results of our study and previous research suggest that physiological stress indicators, mainly heart rate variability measures, are less vulnerable to day-to-day variation than single-point steroid hormone measurements.

Strengths of the study include test-retest, within subject design, and detailed examination of the HPA axis activity since we measured three steroid hormones (cortisol, cortisone, DHEA) and their ratios. However, some limitations should be considered. Firstly, to evaluate the activity of ANS, we employed only time-domain HRV measures (RMSSD, pNN50), which mainly represents the parasympathetic vagal tone. Thus, future research should explore a broader range of HRV indices, including frequency-domain and non-linear measures reflecting sympathetic and parasympathetic branches of the ANS. Secondly, we have utilized salivary cortisol to cortisone ratio as an indirect measure of 11β-HSD2 activity. Evidently, direct and more precise measurement of 11β-HSD2 enzymatic activity would be more preferable as it would bring the possibility to adjust cortisol levels according to 11β-HSD2 activity and thus would lead to better evaluation of the HPA axis and its associations with ANS. Thirdly, due to the observational study design, we cannot determine the direction of causality in relationships between HRV measures and adrenal steroid hormone levels. On the other hand, we expect that a bidirectional association exists between the HPA axis and ANS.

## Conclusions

In this study, we found that higher hypothalamic-pituitary-adrenal axis activity is associated with lower parasympathetic vagal tone under baseline conditions in apparently healthy non-stressed or moderately stressed adults. Due to a positive association between salivary dehydroepiandrosterone level, dehydroepiandrosterone to cortisol ratio, and heart rate variability indices, our results suggest that besides glucocorticoids, androgens might also play an essential role in the molecular mechanisms underlying the linkage between hypothalamic-pituitaryadrenal axis and autonomic nervous system.

## List of abbreviations

ANS, autonomic nervous system; DHEA, dehydroepiandrosterone; DHEA-S, dehydroepiandrosterone sulphate; HPA, hypothalamic-pituitary-adrenal axis; HR, heart rate; HRV, heart rate variability; ICC, intraclass correlation coefficient; PFC, prefrontal cortex; pNN50, percentage of R-R intervals with more than 50-ms variation; PSS, Perceived Stress Scale; RMSSD, square root of the mean squared differences of successive R-R intervals; saCORT, salivary cortisol; STAI, State-Trait Anxiety Inventory; UHPLC, ultra-high performance liquid chromatography; 11β-HSD2, 11β-hydroxysteroid dehydrogenase type 2.

## Acknowledgments

This work was supported by the 2014–2020 European Union Investment in Lithuania (grant number S-J05-LVPA-K-03-0059).

## Conflict of interest statement

The authors stated that they have no conflicts of interest regarding the publication of this article.
